# An open-source framework for synthetic post-dive Doppler ultrasound audio generation

**DOI:** 10.1371/journal.pone.0284922

**Published:** 2023-04-27

**Authors:** David Q. Le, Andrew H. Hoang, Arian Azarang, Rachel M. Lance, Michael Natoli, Alan Gatrell, S. Lesley Blogg, Paul A. Dayton, Frauke Tillmans, Peter Lindholm, Richard E. Moon, Virginie Papadopoulou

**Affiliations:** 1 Joint Department of Biomedical Engineering, North Carolina State University, and The University of North Carolina at Chapel Hill, Chapel Hill, North Carolina, United States of America; 2 Center for Hyperbaric Medicine and Environmental Physiology, Duke University Medical Center, Durham, North Carolina, United States of America; 3 Department of Emergency Medicine, University of California, San Diego, California, United States of America; 4 SLB Consulting, Cumbria, United Kingdom; 5 Divers Alert Network, Durham, North Carolina, United States of America; Ataturk University Faculty of Medicine, TURKEY

## Abstract

Doppler ultrasound (DU) measurements are used to detect and evaluate venous gas emboli (VGE) formed after decompression. Automated methodologies for assessing VGE presence using signal processing have been developed on varying real-world datasets of limited size and without ground truth values preventing objective evaluation. We develop and report a method to generate synthetic post-dive data using DU signals collected in both precordium and subclavian vein with varying degrees of bubbling matching field-standard grading metrics. This method is adaptable, modifiable, and reproducible, allowing for researchers to tune the produced dataset for their desired purpose. We provide the baseline Doppler recordings and code required to generate synthetic data for researchers to reproduce our work and improve upon it. We also provide a set of pre-made synthetic post-dive DU data spanning six scenarios representing the Spencer and Kisman-Masurel (KM) grading scales as well as precordial and subclavian DU recordings. By providing a method for synthetic post-dive DU data generation, we aim to improve and accelerate the development of signal processing techniques for VGE analysis in Doppler ultrasound.

## Introduction

Decompression sickness (DCS) can occur after the human body experiences depressurization leading to the formation of gas bubbles. The formation and growth of these bubbles are understood to be the primary mechanism of DCS through a complex physiological cascade inducing symptoms ranging from skin rash to neurological degradation, and even death [1, 2].

Scuba divers breathe pressurized gas mixtures, of which the inert gases may saturate the tissues at depth. During ascent, the pressure gradient reverses leading to ‘off-gassing’ of supersaturated tissues and generation of bubbles also known as venous gas emboli (VGE). Controlled ascent, often using decompression ‘stops’, is used to mitigate DCS by allowing lung filtration to remove any evolved gas from the blood pool without excessive bubble retention [3]. These ascent profiles are determined using decompression models, which use DCS outcomes as the measurable endpoint. Due to the low incidence of DCS (<1%), decompression algorithm development requires large sample sizes that are not always feasible to provide [4, 5]. Therefore, VGE detection using Doppler ultrasound (DU) has been proposed as a supplemental endpoint for DCS modeling and DCS assessment.

Doppler ultrasound functions by transmitting an acoustic wave and receiving the backscattered signal. Ultrasound reflected off moving scatterers produces a frequency shift in the received signal that falls within the auditory range and thus is typically recorded as a one-dimensional audio signal [6, 7]. Due to the high acoustic impedance mismatch between gas and liquid, VGE are highly echogenic, producing distinct sounds that can be distinguished from cardiac motion or blood flow, and are often described as a ‘ping’ or ‘chirp’. The amount of VGE load post-dive is scored commonly using the Spencer (Table 1) and Kisman-Masurel (KM) (Table 2) grading scales by a trained rater [7–9]. An increasing level of VGE grade has been associated with increased DCS risk, making ultrasound monitoring a valuable tool in assessing human response to diving [10].

**Table 1:** Definition of the Spencer code used for assessing venous gas emboli in post-dive Doppler ultrasound recordings, reproduced from [7].

Spencer Score	Description
**0**	Complete lack of bubble signals
**1**	Occasional bubble signal discernible with the cardiac motion signal, with majority of cardiac periods free of bubbles
**2**	Many but less than half of the cardiac periods contain bubble signals, singularly or in groups
**3**	All of the cardiac periods contain showers or single bubble signals, but not dominating or overriding the cardiac motion signals
**4**	Maximum detectable bubble signal sounding continuously throughout systole and diastole of every cardiac period, and overriding the amplitude of the normal cardiac signals

**Table 2:** Definition of the Kisman-Masurel code used for assessing venous gas emboli in post-dive Doppler ultrasound recordings, reproduced from [7].

KM Score	Bubbles per cardiac cycle	Percentage of cardiac cycles at rest with detectable bubbles	Number of cardiac cycles with bubbles after motion	Amplitude
**0**	0	0%	0	No bubbles discernible
**1**	1–2	1–10%	1–2	Barely perceptible
**2**	Several, 3–8	10–50%	3–5	Moderate amplitude
**3**	Rolling drumbeat > 9	50–99%	6–10	Loud
**4**	Continuous sound	100%	10+	Maximal

Despite its common application in decompression research, manual rating of DU audio is time-consuming, dependent on training, and subject to inter- and intra-rater variability [11]. Automated analysis methods for post-decompression Doppler ultrasound have been previously developed, however progress is inhibited by a lack of standardized datasets allowing for algorithm development, evaluation, and comparison [12, 13].

Recently, a dataset of post-dive Doppler recordings was released for the purposes of automated VGE extraction and grading algorithm development [14]. Several signal-separation algorithms have been explored using this data such as adaptive empirical mode decomposition and complete ensemble empirical mode decomposition [15, 16]. Even with its clear usefulness for the development of new VGE analysis methodologies, this real-world dataset from Pierleoni et al. (2019) still exhibits certain limitations, such as limited dataset size (30 recordings) and use of a fetal Doppler system (FD1 2-MHz Doppler probe, Huntleigh Ltd, Cardiff, UK) which is not standard for all decompression research. The limited dataset recordings only provide grades that range from 0–2.5 on the Extended Spencer Scales, missing out on higher grades of VGE that relate to greater DCS risk. Furthermore, the data labels are of an ordinal scale produced by human graders, thus no ground truth is available providing separate VGE and cardiac signals. This requires algorithm assessment to be performed based on grade prediction accuracy which can be subject to bias, exacerbated by the limited data size.

These issues presented are not unique to post-dive DU analysis but are also found in other physiological signal measurements. Fetal electrocardiograms (fECG) and phonocardiograms (fPCG) are often used to assess fetal heart rate analysis but are obscured by mixing of maternal cardiac signal and background noise. Similarly, signal-separation techniques are not easily assessed in these modalities due to a lack of ground truth to evaluate the extracted fetal signal quality. As such, synthetic data has been proposed to allow for the direct comparison of signal processing methodologies. In fECG, fetal cardiac data is simulated using physiological modeling of cardiac function mimicking sensor placement and noise sources [17, 18]. The synthetic data from this simulation model has been used for signal separation comparisons [17] and the training of deep learning models which require large quantities of samples to prevent overfitting to real-data [19, 20].

Doppler ultrasound of blood flow can be simulated using numerical models to produce pulsatile data [21, 22]. However, cardiac sounds from precordial DU as well as bubble signals have not been modeled making direct simulations of post-decompression data not feasible. In our previous work, we developed a novel deep learning approach for automated Doppler classification trained initially on synthetic data and fine-tuned on real-data [23]. Due to the complexities in fully simulating relevant and accurate synthetic DU data, we procedurally combined experimentally collected human cardiac and flowing bubble DU audio creating data spanning all 5 Spencer grades. Trained solely on real-world data (274 recordings), the proposed deep learning network was only able to achieve 60.3% average ordinal accuracy in precordial DU and 64.2% subclavian DU average ordinal accuracy. However, network pretraining using synthetic data prior to fine-tuning with real data improved the network performance to 84.9% and 90.4% for precordial and subclavian DU classification, respectively.

Here we describe and reiterate the methods for synthetic data generation described in our previous work, while expanding the types of data being generated. In particular, we augment the precordial baseline data with the addition of another 3 non-diving subjects, add subclavian baseline data from previously acquired pre-dive data from 75 subjects in the field, change our superposition algorithm to allow for bubbles both in-between cardiac cycles and throughout the cardiac cycle, and generate datasets for both KM and Spencer grades in each case. We additionally provide all codes and data, consisting of 15,000 Spencer and 66,000 KM 10-second synthetic audio recordings, to aid in the development and evaluation of automated DU VGE assessment methods.

## Materials and methods

### 1. Baseline data collection

#### 1.1 Baseline precordial data collection

The baseline non-diving precordial data used in this work is the same as in (21), with the exception of additional datasets from 3 subjects and removal of 2 subjects that produced relatively lower quality data (total of 16 subjects vs 15 previously). Briefly, precordial DU recordings were collected in 16 healthy human compensated volunteers (age 18–50) and free of heart murmurs who had given informed consent. The study was approved by the Duke Health Institutional Review Board (Pro#00105294). Precordial DU was performed on either or both clinical DU scanners to replicate baseline recordings used in historical ultrasound database. Volunteers were measured initially at rest, then with leg flexions. Continuous-wave (CW) Doppler ultrasound was obtained using a DBM9008 Doppler Bubble Monitor system (Techno Scientific, Inc., Concord, Ontario) with a TSI-DPA7 2.5 MHz continuous wave precordial transducer. Pulse-wave (PW) ultrasound measurements were obtained using the DBM9610 Doppler Bubble Monitor (Techno Scientific, Inc. Concord, Ontario) with the same transducer. Doppler audio signals were recorded from the systems using an Analog-to-Digital converter (Behringer, U-control UCA222) and recorded to a laptop using Audacity software (v2.4.2, Audacity Team, USA) at 44.1 kHz sampling frequency. Eight subjects were measured with both CW and PW systems, and eight were measured using only the CW system. Each recording ranged between 3:30 to 7 minutes.

#### 1.2 Baseline subclavian data

Previously collected, de-identified, subclavian ultrasound Doppler data were also used. These were originally collected on 75 adult scuba diving volunteers with a minimum of 50 logged dives and “advanced open water diver” certification (predive and every 20 min after surfacing from unrestricted dives for at least 1 h) after informed consent and as approved by the DAN institutional review board (#024-19-22) [24]. Here only pre-dive recordings are used (to guarantee no bubbles). Subclavian Doppler ultrasound was performed using the O’Dive^™^ continuous wave Doppler device (Azoth, France) and recorded on an iPad at 48 kHz sampling frequency. Measurements were performed on both left and right subclavian veins, at rest. Each recording ranged from 18–20 seconds.

#### 1.3 Baseline bubble data collection

The method for generating and collecting single-bubble Doppler recordings is described elsewhere [23]. Briefly described, a tissue-mimicking flow phantom was fabricated by first dissolving 85% distilled water with 10g/mL porcine gelatin (Gel strength 300, type-A, Sigma-Aldrich, St. Louis, MO, USA), and 5% 1-propanol (Fisher Chemical, Hampton, NH, USA). The ingredients were mixed in a beaker with a stir bar over a hot stir plate and heated to 60°C for 30 minutes. The mixture was then placed under vacuum for 30 minutes for degassing, then poured into a custom mold with an 8.9 mm rod passing through the center. After setting, the rod was removed, leaving a wall-less vessel. A peristaltic pump (INTLLAB, Shenzhen, CN) was connected to the phantom vessel inlet and outlet, with a reservoir of water to allow for bubbles to dissipate without recirculation. The pump was controlled using an Arduino Due (Arduino, Somerville, MA, USA) at flow rates ranging from 540–900 mL/min.

**Fig 1:**
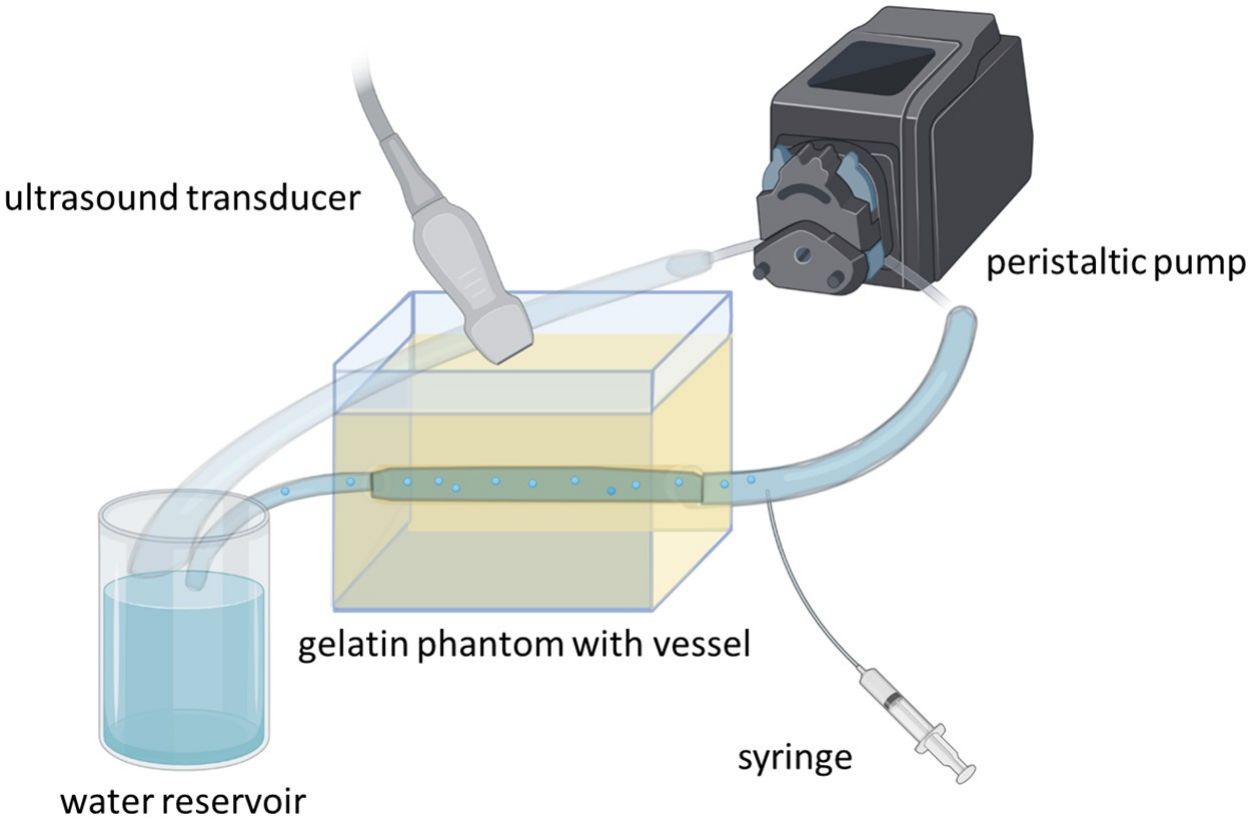
Schematic of the experimental setup used for laboratory Doppler ultrasound bubble recordings. A peristaltic pump was used to provide a water flow through a wall-less vessel situated in a gelatin phantom, where air bubbles were injected and measured with Doppler ultrasound above the phantom at different angles.

Doppler ultrasound measurements were acquired using a Siemens/Acuson Sequoia C512 (Mountain View, CA, USA) with an L11-5 transducer positioned along the vessel and set to pulse-wave Doppler mode (7 MHz). The Doppler audio output from the US machine was converted to a digital signal using an off-the-shelf analog-to-digital converter and recorded using Audacity (v2.4.2, Audacity Team, USA) with a sampling frequency of 44.1 kHz. For the experiment, the pump was set to 540, 720, and 900 mL/min, the transducer was placed at one of three angles relative to the water flow (75°, 90°, and 105°). A 24g catheter was inserted along the tubing near the phantom entrance connected to a 20 mL air-filled syringe (Becton-Dickinson, Franklin Lakes, NJ, USA). Air from the syringe was injected into the flowing water using a syringe pump (Harvard Apparatus, Holliston, MA, USA) with an injection rate of 0.1 mL/min to produce individual bubbles that were audibly separable. A graphical representation of the flow-phantom is provided in Fig 1.

For each parameter combination, 5 audio recordings of 25-second duration were acquired, producing 45 recordings. An additional experiment was performed with the pump flow randomly varying between 540–900 mL/min at each specified angle described previously, producing 15 additional recordings.

### 2. Data preprocessing and synthetic data generation

All work presented was developed and executed in MATLAB 2021b (Mathworks, Natick, MA, USA).

#### 2.1 Data preprocessing

For the human baseline dataset, the audio recordings were annotated on one channel while the Doppler data was recorded on the second. The annotations were automatically removed by calculating the mean of the envelope-detected channel-data and retaining the channel with the greatest energy. The precordial baselines were additionally manually cleaned by removing sections that contained no audio or major artifacts such as motion. Then, the audio files are resampled from the native sampling frequency (i.e. 44.1kHz) to a desired frequency (i.e. 8 kHz) for efficient memory usage.

Experimental Doppler bubble recordings were segmented for individual bubble signals. This was performed by performing peak detection on the envelope detected signal. The full-width 20% max amplitude of the peak was used to segment the whole bubble signal. Furthermore, all segmented signals longer than 0.5 seconds were removed from the dataset.

#### 2.2 Synthetic data generation procedure

Synthetic post-dive DU data was generated using the following process, originally proposed in Azarang et al. 2022 [23], but is expanded to include subclavian data as well as selective placement of VGE in between cardiac cycles without cardiac interference. We initially select one of the available human baseline recordings, and then segment the audio to the desired length with an added 50% sample buffer at a random location along the signal. This audio signal is then transformed using a time-series audio data augmenter available in MATLAB version 2019b and newer. During this process, four types of augmentation are applied: 1) time-stretch with a speed-up factor (0.8–1.3), 2) pitch shift between -2 and 2 semitones, 3) volume gain between -10 and 10 dB, and 4) time-shift with a range of the entire length of the signal. These augmentations introduce additional variability not available in the baseline data collected and serve to increase the representation of conditions that may affect the sound of cardiac Doppler recordings such as heart rate, varying background noise, and sensitivity.

After augmentation, cardiac cycles must be detected to allow for the placement of bubbles into a percentage of the audible cardiac cycles. Envelope detection is performed on the augmented cardiac signal and normalized. Subsequently, heart rate estimation using autocorrelation [25] is extracted from this signal and used as a parameter in determining the total number of cardiac cycles and distance between peaks for the peak detection function provided in MATLAB. All heart cycles are defined as the space between each peak and are stored in an array for use during bubble-only data generation.

Cardiac windows are defined two-fold: 1) Full cycles and 2) partial cycles. The full cycle window allows for bubbles to be placed anywhere from peak-to-peak of a cardiac cycle regardless of cardiac sound amplitude. Using this window definition, VGE that are placed into the cardiac data are occasionally obscured by cardiac noise, producing a more difficult dataset for signal-separation. In contrast, partial cycle cardiac windows only allow for bubble placement in regions where the background noise is 50% of the maximum amplitude in a cardiac cycle. This increases the differentiability between cardiac and bubble signals and may be more representative of what graders will normally hear. Furthermore, partial-cardiac windows may be more applicable to precordial DU as the cardiac sounds are much greater in amplitude compared to subclavian vein measurements and as such, we only apply this windowing technique to our synthetic precordial data [13, 26].

A KM value is initially selected randomly from all possible KM value combinations. The KM scale consists of three values pertaining to three separate parameters used to define the grade assigned and thus the VGE load. We implement a modified KM scale based on the original interchanging descriptive parameters with quantifiable values detailed in Table 3, where *P*_*γ*_ is defined in Eq (1)

$$P_{\gamma }=\frac{Cardiac\;Period\;(s)}{Average\;Bubble\;Length\;\left(s\right)}$$ (1)

and used in calculating the maximum number of bubbles to be considered “rolling” and “continuous” as defined by the KM scale (4).

**Table 3:** Modified Kisman-Masurel code for use in procedural synthetic data generation. *P*_*γ*_ is used in calculating the maximum number of bubbles that can be added to approximate a “rolling” and “continuous” sound in KM grades 3 and 4, as defined in text in Eq (1).

KM Score	Bubbles per cardiac cycle	Percentage of cardiac cycles at rest with detectable bubbles	Relative Amplitude
**0**	0	0%	0.000
**1**	1–2	1–10%	0.100–0.250
**2**	3–8	10–50%	0.250–0.450
**3**	9 *P*_*γ*_	50–99%	0.450–0.775
**4**	*P*_*γ*_ − (2 * *P*_*γ*_)	100%	0.775–0.999

**Fig 2:**
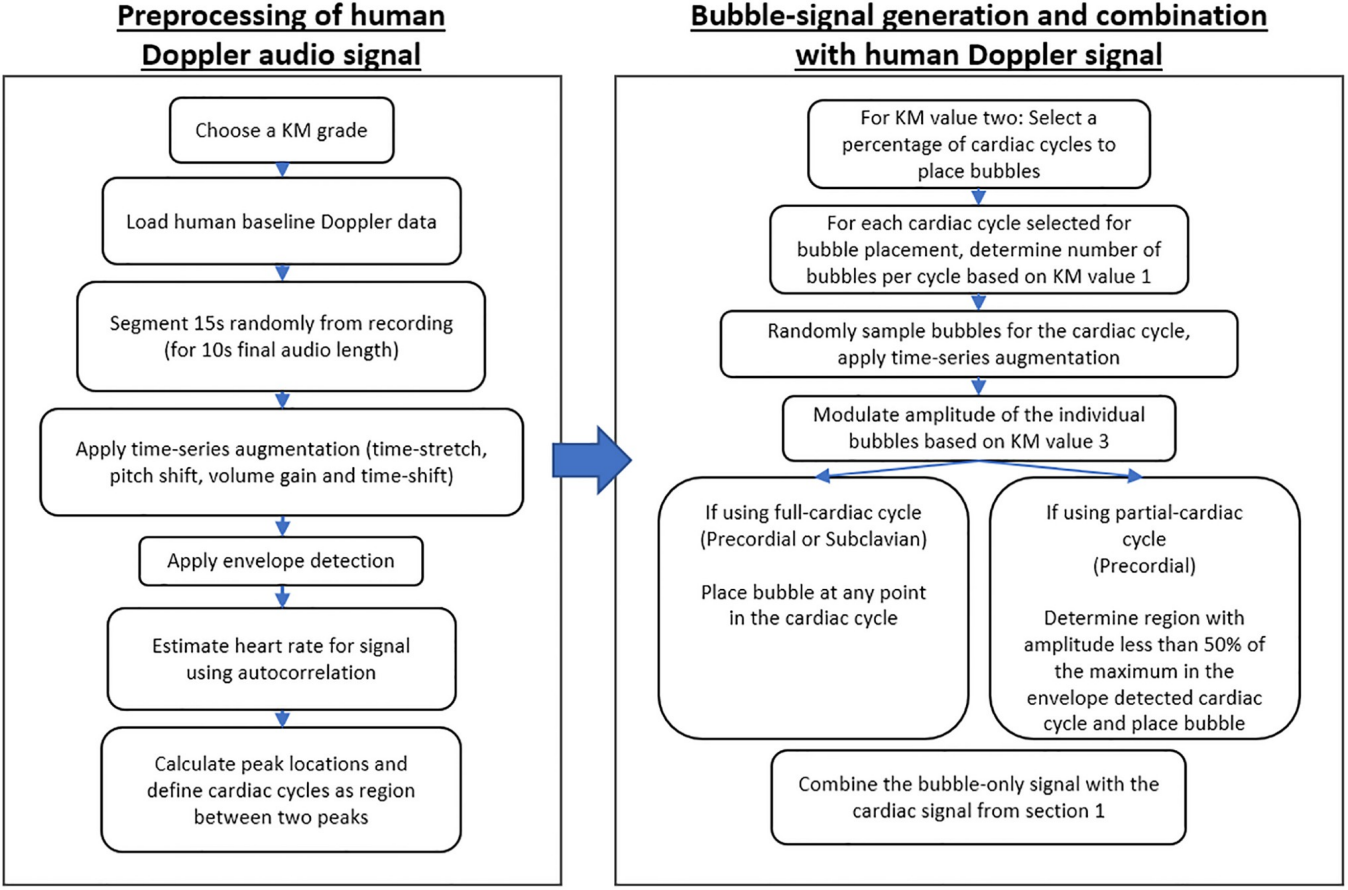
Flow diagram detailing the synthetic data generation process.

The algorithm begins with the second KM value and randomly generates a percentage value that is then multiplied by the total number of cardiac cycles in the cardiac data (Table 3). From there, that number of cardiac cycles are sampled from the total signal. For each cardiac cycle, a random number of bubbles based on the ranges defined on the first KM value in the modified KM scale (Table 3) is sampled from the available bubbles recorded experimentally. Each of these sampled bubbles are individually augmented with time-stretch (range from 0.8–2.0) and pitch-shift between -2 and 2 semitones. These augmented bubbles are then normalized, multiplied by an amplitude value defined by the third KM value (Table 3), amplitude modulated by ±15%, then randomly placed into an array equal length to the cardiac cycle with low-amplitude random Gaussian noise with zero mean. This process is performed for each cardiac cycle selected for bubble-placement until the whole bubble-only signal is generated. A flow-diagram describing these methods is summarized in Fig 2.

**Fig 3:**
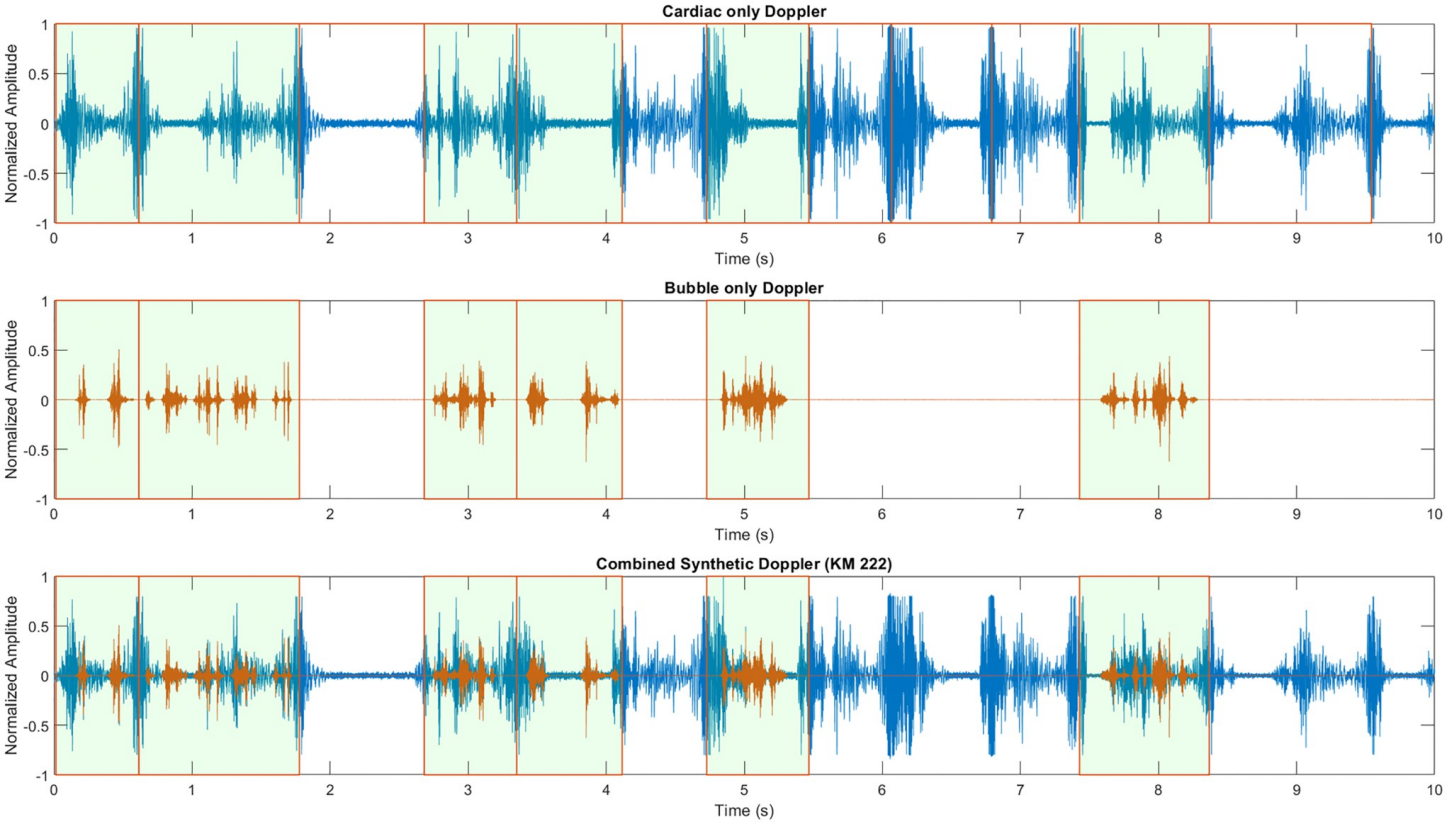
Graphical representation of KM grade (222) synthetic data generation process. A) Original cardiac data with windows showing all cardiac cycles segmented by orange vertical lines. Based on the KM2 value of 2, 50% of the cardiac windows are selected for bubble-placement (6/12). B) For each selected window, a number of bubbles based on KM1 is chosen and placed into that cardiac window, bubble-audio is shown in orange. C) Summed cardiac and bubble-only audios to generate the final synthetic combined audio recording.

The cardiac-only signal and bubble-only signal are then used to create the post-dive synthetic Doppler audio by summation. An example synthetic Doppler recording is shown in Fig 3, corresponding to KM grade 222 using precordial baseline data and full-cardiac cycles for VGE placement. All three signals are then center cropped to the desired length defined by the user and saved with the same name as.wav files in three separate folders. Using the same filename allows for simple matching between the components and synthetic DU audio. Data can be grouped into subfolders based on their KM grade or Spencer grade if converted using the conversion table (Table 4). An example synthetic Doppler recording is shown in Fig 2, corresponding to KM grade 222 using precordial baseline data and full-cardiac cycles for VGE placement.

**Table 4:** Kisman-Masurel to Spencer conversion chart. Conversion chart between Spencer and Kisman-Masurel codes as defined in [27].

Spencer Score	Kisman-Masurel Grades
0	[000]
1	[111] [112] [113] [211] [212] [213]
2	[121] [122] [123] [221] [222] [223]
3	[232] [233] [242] [243] [332] [333] [342] [343]
4	[444]

## Dataset and results

**Fig 4:**
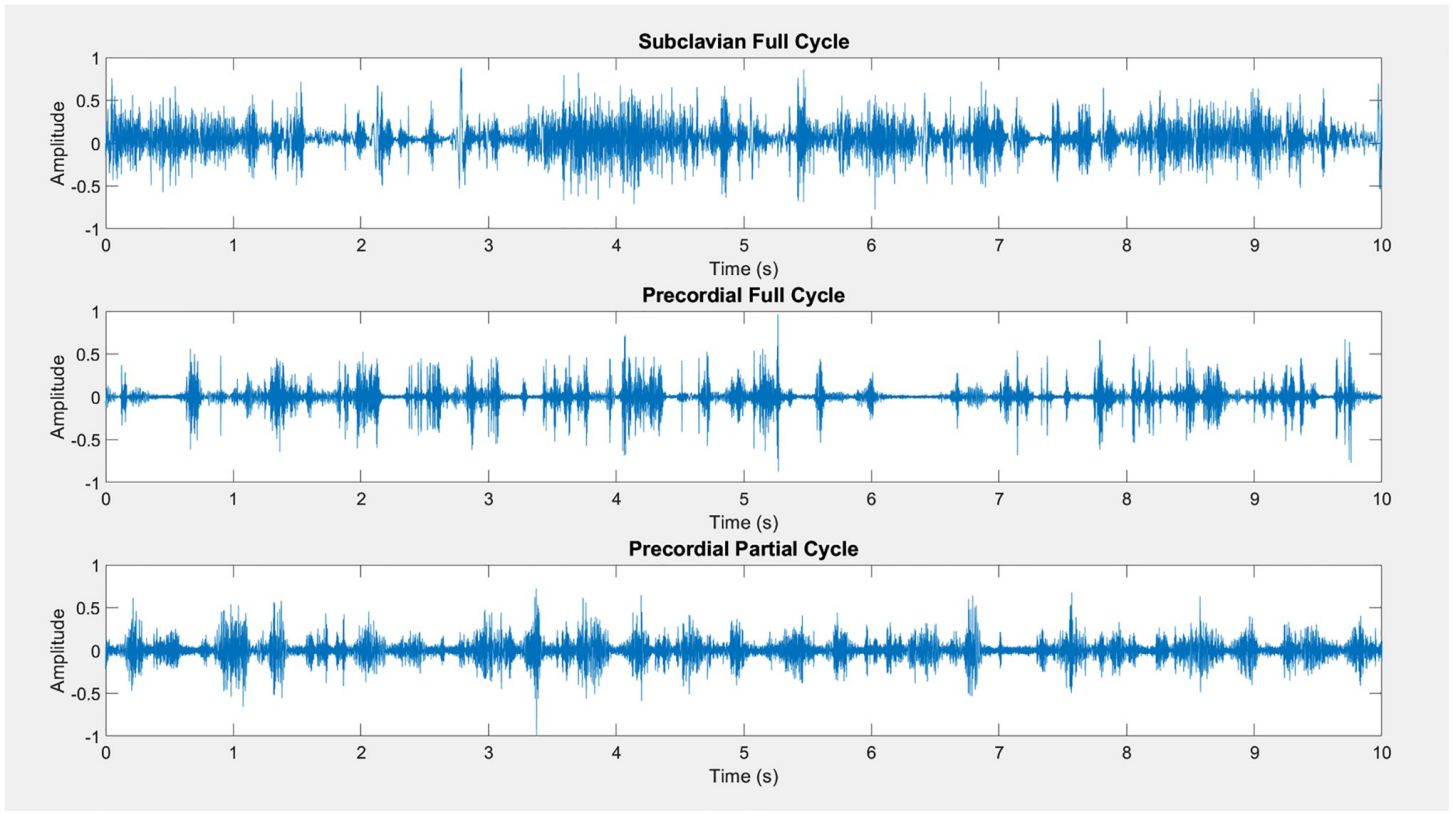
Examples of Spencer grade 4 for 3 conditions: 1) Subclavian data with VGE placed at any point along the signal. 2) Precordial data with VGE placed at any point in the signal, and 3) Precordial data with VGE placed only within the regions with amplitude less than 50% of the maximum amplitude between cardiac cycles.

Using the method described above, we are able to generate a total of six types of data (Table 5), using Spencer or KM classes, precordial or subclavian, and full or partial cardiac cycles for VGE placement. Fig 4 shows examples of Spencer grade 4 post-dive Doppler signals for three cases: 1) Subclavian 2) Precordial with full-cardiac cycle VGE placement, and 3) Precordial with partial-cardiac cycle VGE placement. Only precordial data were generated for the partial-cardiac cycle method as subclavian background noise does not typically obscure VGE signals. The dataset provided contains all baseline precordial and subclavian recordings as well as the isolated VGE DU audio. Furthermore, we include generated data using our algorithm for each of the six scenarios described in Table 5. In the pre-generated data, 1000 10-second samples for each class are provided (5-class for Spencer and 22 classes for Kisman-Masurel as seen in Table 4). This results in the generation of a dataset of 15,000 10-s recordings of Spencer (1000 x 5 grades x 3 [precordial full cardiac cycles, precordial partial cardiac cycles, subclavian]), and 66,000 10-s KM synthetic datasets (1000 x 22 grades x 3 [precordial full cardiac cycles, precordial partial cardiac cycles, subclavian]). Counting the corresponding bubble only and cardiac only signals, a grand total 243,000 audio files is therefore generated and shared. We release all codes and generated data under an open-source license (GNU General Public License v2.0) for use and improvement by others. The code is available on GitHub at https://github.com/dle4/Synthetic-Post-Dive-Ultrasound-Audio-Generator and the dataset on the Dryad repository at https://doi.org/10.5061/dryad.xgxd254kp. A set of example data is also presented as Supplemental material linked to this paper.

**Table 5:** Description of data types that can be generated using the proposed algorithm.

Case	Description
Case 1	Precordial DU, Spencer grading scale, VGE placed into full-cardiac cycles
Case 2	Subclavian DU, Spencer grading scale, VGE placed into full-cardiac cycles
Case 3	Precordial DU, Kisman-Masurel grading scale, VGE placed into full-cardiac cycles
Case 4	Subclavian DU, Kisman-Masurel grading scale, VGE placed into full-cardiac cycles
Case 5	Precordial DU, Spencer grading scale, VGE placed into low-amplitude cardiac regions
Case 6	Precordial DU, Kisman-Masurel grading scale, VGE placed into low-amplitude cardiac regions

**Fig 5:**
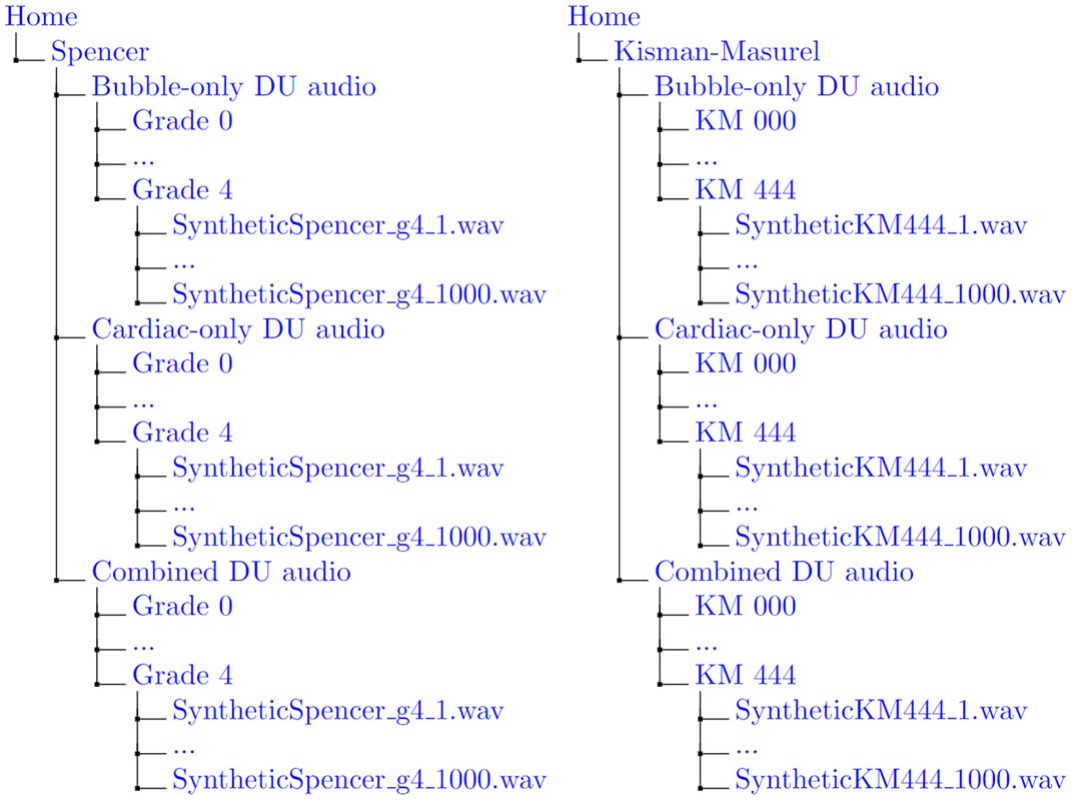
Directory tree representation of directory system containing the generated synthetic data upon execution.

The generated data is organized into six directories for each of the cases described in Table 5. For each case, three sub-directories are available, denoting the isolated cardiac-only audio, isolated bubble-only audio, and combined synthetic post-dive audio. Within each of those directories, the data is further organized by VGE grades (Spencer grade 0–4 or KM grade 000–444). Within each of those sub-directories exists the generated DU audio file, with naming convention “SyntheticGeneratorCase_classLabel_#.wav”. For each audio file in the combined directory, the isolated human and VGE DU recordings used to generate the synthetic data can be found with the same filename in the parallel directory paths. The identical naming convention allows for simple reference to the audio files used to generate the final synthetic DU file. A directory tree representation of the code output is presented in Fig 5. This directory path system can be user-modified as desired for their intended purposes.

Using this algorithm, a single 10-second Doppler audio file is generated in 0.1816 seconds using a computer with an Intel core i9-9940x averaged over 100 files. Using parallelization, 14 recordings can be generated simultaneously per iteration, significantly improving the data generation rate.

## Discussion

Due to the limited availability of real-world post-dive DU data, we have developed a method for generating synthetic DU audio capable of representing the large degree of variability found *in vivo*. We do not intend for this dataset to be a replacement for real-world data, but to be used as supplemental data for the development of automated VGE analysis methods. Using the framework presented here, it is possible to generate an unlimited number of samples and it can be modified further by others to allow for recordings of longer length, greater noise, or different VGE grading scale definitions.

Furthermore, due to the separate sources of bubble-only and cardiac-only data, signal separation methods may be evaluated upon this data for proof-of-concept validation as well as performance comparison with other techniques. Simulated fetal echocardiography data generated in other studies [18, 28] have been used for this purpose as an initial benchmark for various fECG extraction algorithms. Additionally, compared to the sole use of Spencer grade classification accuracy as the final metric for DU VGE analysis, the ground truth bubble-only DU data containing the exact number and placement of bubbles is available to researchers, and can be used for more detailed evaluation metrics. For example, a subset of the synthetic data presented here has been used in our prior work, enhancing the performance of a deep-learning algorithm tasked with Spencer grade classifications [23]. Additional validation of this dataset as a benchmark for signal separation or other VGE classification systems could be envisaged in future work.

Several limitations for the synthetic data generator are present in this work. One of these limitations is that the data generated is, in the case of precordial recordings, relatively clean and free from motion artifacts and noise that may be present in real-world situations. This occurs due to the baseline data being collected in a controlled laboratory setting as well as the algorithm itself relying on cardiac cycle detection which fails when motion artifacts occur in the source data. Additionally, data variability is limited based on the source of the baseline human and VGE data. In this work, we collect human baseline data using clinical and commercial devices that are not always used in the research field and attempt to address this using audio-based time-series augmentation to modify the data available. However, the dataset provided by Pierleoni et al. (2019) used a fetal Doppler system which may have different signal processing and output compared to our systems that may not be replicated using this augmentation strategy. This can be remedied by the addition of baseline data using other devices and by the inclusion of additional individuals to generate a more representative dataset. Finally, our data has only been evaluated qualitatively by our expert DU operator (SLB) and although found to be similar to real-world data, is not guaranteed that a certain grade will be identical to a trained rater. The algorithm provided follows the KM scale definitions with minimal interpretation, however, due to the variability between trained raters as described in [11] it is difficult to find one set of parameters that will perfectly represent all raters. As such, the parameters provided for our modified KM scale can be changed by the end user.

## Conclusion

The post-dive DU audio synthetic data generator presented in this work allows for researchers to create DU data across a large spectrum of real-world cases for example: precordial or subclavian measurements; at rest or flex; with and without strong distinction between VGE and cardiac noise; and KM or Spencer VGE grading systems. Using this generator, the user can be provided with the isolated ground truths for the human and VGE signal components for each synthetic DU audio which can be aid in the validation of VGE extraction techniques of relevance to decompression research. Furthermore, this synthetic data can be used to supplement real-world data for deep-learning approaches to VGE analysis. We release all codes under an open-source license for use and improvement by users, available at https://github.com/dle4/Synthetic-Post-Dive-Ultrasound-Audio-Generator, as well as 243,000 audio files comprising synthetic Doppler ultrasound signals and their corresponding bubble and cardiac only signals on https://doi.org/10.5061/dryad.xgxd254kp.

## Supporting information

S1 Text We provide a small subset of the full synthetic dataset as supplemental data at https://doi.org/10.5061/dryad.xgxd254kp. These data cover the six cases described in Table 5, with one recording per VGE grade; following the same directory structure as described in the dataset section.
(TXT)

S1 File (ZIP)
